# Nutritional Support in Cancer patients: update of the Italian Intersociety Working Group practical recommendations

**DOI:** 10.7150/jca.73130

**Published:** 2022-05-21

**Authors:** Riccardo Caccialanza, Paolo Cotogni, Emanuele Cereda, Paolo Bossi, Giuseppe Aprile, Paolo Delrio, Patrizia Gnagnarella, Annalisa Mascheroni, Taira Monge, Ettore Corradi, Michele Grieco, Sergio Riso, Francesco De Lorenzo, Francesca Traclò, Elisabetta Iannelli, Giordano Domenico Beretta, Michela Zanetti, Saverio Cinieri, Vittorina Zagonel, Paolo Pedrazzoli

**Affiliations:** 1 Clinical Nutrition and Dietetics Unit, Fondazione IRCCS Policlinico San Matteo, Pavia, Italy; 2 Pain Management and Palliative Care, Department of Anesthesia, Intensive Care and Emergency, Molinette Hospital, University of Turin, Turin, Italy;; 3 Medical Oncology Unit, ASST Spedali Civili di Brescia, and Department of Medical and Surgical Specialties, Radiological Sciences, and Public Health, University of Brescia, Brescia, Italy; 4 Department of Oncology, San Bortolo General Hospital, Vicenza, Italy; 5 Colorectal Surgical Oncology-Abdominal Oncology Department, Istituto Nazionale per lo Studio e la Cura dei Tumori, Fondazione Giovanni Pascale IRCCS, Naples, Italy; 6 Division of Epidemiology and Biostatistics, IEO European Institute of Oncology IRCCS, Milan, Italy; 7 Clinical Nutrition and Dietetics Unit, ASST Melegnano-Martesana, 20077 Melegnano (MI), Italy; 8 Clinical Nutrition Unit, S. Giovanni Battista Hospital, Torino, Italy; 9 Clinical Nutritional Unit, ASST Grande Ospedale Metropolitano Niguarda, Milano, Italy; 10 Department of Surgery, Sant' Eugenio Hospital, Rome, Italy; 11 Clinical Nutrition and Dietetics Unit, Maggiore della Carità Hospital, Novara, Italy; 12 Italian Federation of Volunteer-based Cancer Organizations, Rome, Italy; 13 Department of Oncology, Humanitas Gavazzeni, Bergamo, Italy; 14 Department of Medical, Surgical and Health Sciences - University of Trieste, and Azienda Sanitaria Universitaria Giuliano Isontina (ASUGI), Trieste, Italy; 15 Medical Oncology Division and Breast Unit, Senatore Antonio Perrino Hospital, ASL Brindisi, Brindisi, Italy; 16 Oncology Unit 1, Department of Oncology, Veneto Institute of Oncology-IRCCS, 35128 Padova, Italy; 17 Medical Oncology Unit, Fondazione IRCCS Policlinico San Matteo and Department of Internal Medicine, University of Pavia, Pavia, Italy

**Keywords:** nutritional support, cancer patients, malnutrition, practical recommendations, nutritional care

## Abstract

Malnutrition is a frequent problem in cancer patients, which leads to prolonged and repeated hospitalizations, increased treatment-related toxicity, reduced response to cancer treatment, impaired quality of life, a worse overall prognosis and the avoidable waste of health care resources.

Despite being perceived as a limiting factor in oncologic treatments by both oncologists and patients, there is still a considerable gap between need and actual delivery of nutrition care, and attitudes still vary considerably among health care professionals.

In the last 5 years, the Italian Intersociety Working Group for Nutritional Support in Cancer Patients (WG), has repeatedly revisited this issue and has concluded that some improvement in nutritional care in Italy has occurred, at least with regard to awareness and institutional activities. In the same period, new international guidelines for the management of malnutrition and cachexia have been released.

Despite these valuable initiatives, effective structural strategies and concrete actions aimed at facing the challenging issues of nutritional care in oncology are still needed, requiring the active participation of scientific societies and health authorities.

As a continuation of the WG's work, we have reviewed available data present in the literature from January 2016 to September 2021, together with the most recent guidelines issued by scientific societies and health authorities, thus providing an update of the 2016 WG practical recommendations, with suggestions for new areas/issues for possible improvement and implementation.

## Introduction

Although malnutrition is recognized by both oncologists and patients as a limiting factor in oncologic treatments, it remains poorly managed 1. The consequences are serious, leading to reduced anticancer treatment tolerance, poorer prognosis, impaired quality of life (QoL) and the avoidable waste of health care resources associated with prolonged and repeated hospitalizations 2. Nevertheless, adherence to international guidelines and recommendations is still low, which limits access to high quality nutrition therapy both during and following cancer treatment 3.

Despite the abundance of scientific literature highlighting the problem, and the availability of international guidelines for managing nutritional care in cancer patients, many patients do not receive adequate nutritional support 2-4. Beyond the obvious clinical consequences, overlooking nutrition care incurs billions in healthcare costs 5-8.

The Italian Association of Medical Oncology, the Italian Society of Artificial Nutrition and Metabolism and the Italian Federation of Volunteer-based Cancer Organizations implemented in 2016 a collaborative Working Group (WG) and initiated a structured project named “Integrating Nutritional Therapy in Oncology”, with the aim to increase the awareness of nutritional issues among oncologists and, consequently, to improve the nutritional care of cancer patients in Italy 9. In 2019, the Italian Society of Surgical Oncology and the Technical Scientific Association of Food, Nutrition and Dietetics joined the WG, which was named “Italian Intersociety Working Group for Nutritional Support in Cancer Patients”.

Among its activities, in 2016 the WG issued the first inter-society consensus document in order to provide suitable, concise and practical recommendations for appropriate nutrition in cancer patients 10. This publication was not meant to be a surrogate for international guidelines, but its aim was to provide oncologists, other professionals involved in cancer care and the patients themselves, with a concise, easily accessible and updated summary of the main recommendations needed to appropriately manage nutritional care in oncology.

In the last 5 years, several further initiatives have been undertaken by the WG 11, which has concluded that some improvement in nutritional care in Italy has occurred, at least as far as awareness 2 and institutional practices are concerned 12. In the same period, new international guidelines for the management of malnutrition and related syndromes - such as cachexia - have been released 13-15.

While this represents progress, nutritional care in oncology is still inadequate and needs the involvement and cooperation of scientific societies, the Ministry of Health and the Ministry of Education. Consequently, the WG decided to update the 2016 recommendations, which are presented here. The aim of this document is to: 1) stimulate the national and international Oncology Scientific and Clinical Community; 2) to increase the awareness on nutritional care; 3) to improve the clinical nutrition management of patients with cancer through the provision of simple but mandatory nutrition protocols for daily oncological practice.

## Methodology

The WG included physicians (nutrition specialists, oncologists and surgeons), dietitians and patient representatives. We reviewed available data on the nutritional management of patients with cancer, which appeared in the literature from January 2016 to September 2021, including the evidence-based recommendations released in the guidelines issued by scientific societies and health authorities. Authors were also asked to identify further references from their personal collection of literature or other sources and to choose the most relevant ones to be included in the manuscript. After critical evaluation of literature, the original 2016 WG recommendations have been implemented along with accompanying commentaries. Compared to the 2016 paper, we chose to modify the structure, focusing still on nutritional risk and malnutrition recognition, nutritional counseling and oral supplementation, but then, also, on the different phases of the disease, together with current critical issues and future perspectives.

The drafting process was based on a consensus discussion followed by Delphi rounds and votes until agreement was reached. A final version of the paper was circulated and approved by the scientific board of the endorsing scientific societies, which exclusively funded the present project.

## Early Recognition of Nutritional Risk and Malnutrition

Screening is key to identifying the risk of malnutrition 16. If nutrition risk is not assessed at the first oncologic visit, nutritional deficiency will be missed in half the patients, and appropriate measures to counteract it will not be implemented 17,18.

A number of techniques have been used to assess nutrition status in cancer patients although no 'gold standard' has emerged as superior for sensitivity or specificity. The most frequently employed tools are: the Nutritional Risk Screening 2002 (NRS 2002), the Malnutrition Universal Screening Tool (MUST), the Malnutrition Screening Tool (MST), the patient-generated subjective global assessment (PG-SGA), and the Mini Nutritional Assessment (MNA) 17.

They all showed a moderate to substantial agreement with one another and should be employed as tools to guide corrective measures. There is no comprehensive evaluation of their comparative predictive and/or prognostic value on patient outcomes 19.

More recently, the Global Leadership Initiative on Malnutrition (GLIM) criteria, based on a consensus of experts, provides a diagnostic and operational tool to identify and treat malnutrition in several settings 20. They consider phenotypic and etiological criteria and could be helpful in sharing standardized data worldwide.

Independently of the selected criteria/parameters, nutritional status should be considered a dynamic concept, particularly in oncology; therefore, nutritional screening tests should be administered early and periodically repeated, preferably by nurses, during the whole of the patient's journey - at each outpatient visit and within 48 hours of hospital admission.

As stated by all the available guidelines and recommendations, patients at risk of malnutrition should be referred to a clinical nutrition service/unit/professional for nutritional assessment and treatment. However, due to the foreseeable clinical course, it is reasonable to suggest that patients with certain cancer type (head&neck [H&N], gastrointestinal [GI], lung), advanced disease stage or undergoing more aggressive treatments (high-dose chemotherapy [CT], radical radiotherapy [RT], major abdominal surgery or multimodal [either combined or sequential]), all of which are expected to affect nutritional status, should be immediately referred to clinical nutrition specialists for early comprehensive nutritional assessment, counseling/support and a strict monitoring program, independently of risk evaluation.

The assessment of nutritional status should preferably include tools to identify both malnutrition and to measure body composition, with particular reference to sarcopenia and muscle mass determination 20-25.

The nutritional evaluation should include the combination of different parameters 20: anthropometric measurements (body weight, height, body mass index [BMI]), unintentional weight loss enquiry, biochemical data related to metabolic and inflammatory status, the assessment of nutritional intake, QoL, and physical function tests (gait speed, grip strength) to assess muscle performance 21.

Scientific literature suggests that the exclusive use of anthropometric measures is not sufficient to identify body composition alterations, particularly with respect to muscle mass loss 24. Body composition assessment in cancer patients can be performed by Dual-Energy X-ray Absorptiometry (DEXA) or Bioelectrical Impedance Vectorial Analysis (BIVA), the latter also providing information on hydration and cell mass integrity 26. In particular, low phase angle is a predictor of compromised nutritional status, impaired muscle function, increased risk of morbidity, and reduced survival 26,27.

Computed Tomography and Magnetic Resonance Imaging are the gold standard techniques to assess body composition and their imaging of lumbar vertebra L3 correlates well with whole-body skeletal muscle mass 22,28.

## Nutritional Counseling and Oral Supplementation

Nutritional support should be provided to malnourished patients and those at nutritional risk, in particular when oral energy intake is already insufficient or expected to be inadequate (<60% of estimated caloric requirements) for more than 7 days 13,29,30. The aim of nutritional counseling is to maintain or improve food intake through a diet enriched in calories, proteins and fluids that are better tolerated, and to favour the management of the nutrition impact symptoms (i.e. anorexia, nausea, vomiting, diarrhea, and dysphagia). It should be the first type of support proposed and should be carried out by a dietitian with documented skills in cancer patient care 10,12 for appropriate dietary intervention and its monitoring 31,32. As reported in **Table 1**, this process includes a few steps 33 and aims at providing patients with a thorough understanding of nutritional topics that can lead to long-lasting changes in their eating habits, taking into account individual preferences, ethnicity, culture, estimated nutritional requirements and cancer treatment side effects.

Practical suggestions for managing common symptoms related to cancer treatment, leading to impaired food intake or malabsorption, should be foreseen to optimize patients' diets, in order to cope with nutritional deficiencies and possible swallowing difficulties.

Nutritional interventions should compensate for inadequate energy intake with the objective of improving clinical outcomes. So far, numerous reviews have been published 34-40 in malnourished hospitalized and community-dwelling adults with cancer.

Multiple nutrition interventions have been proposed, including dietary counseling or advice, oral nutritional supplements (ONS) and enteral nutrition (EN). The evidence for nutritional counseling to improve clinical outcomes is heterogeneous. According to the most recent review, nutrition interventions were found able to improve body weight and BMI, nutritional status, protein and energy intake, QoL and response to cancer treatments 40. Inconclusive results were found regarding body composition, functional status, complications, unplanned hospital readmissions and survival. Interestingly, Richards and colleagues found that early nutrition intervention, that is initiated within the first week of cancer treatment, can improve patient prognosis and outcomes 40.

When dietary measures fail to meet patients' protein-calorie requirements as detected by nutritional monitoring, the prescription of energy-dense ONS should be considered, due to their proven efficacy in increasing protein-calorie intake and to fill nutritional gaps 13,41.

In patients with cancer, systemic inflammation inhibits nutrient utilization and promotes catabolism, thus leading to muscle breakdown. Calorie and protein fortification of regular foods, even with standard ONS, does not reduce systemic inflammation. Updated nutritional strategies now suggest considering nutrition with anti-catabolic and inflammation-suppressing ingredients. Studies have indicated that ONS with addition of essential amino acids or high-dose leucine may improve muscle protein synthesis even in the presence of inflammation, although results have not been fully consistent 42,43.

Fish oil, a source of long chain omega-3 fatty acids, is currently suggested to improve appetite, oral intake, lean body mass, and body weight in patients with advanced cancer and at risk of malnutrition 13,44.

The European Society of Clinical Nutrition and Metabolism (ESPEN) guidelines on nutrition in cancer patients recommend supplementation with fish oil, a source of long chain omega-3 fatty acids, to stabilize or improve appetite, food intake, lean body mass, and body weight for patients with advanced cancer undergoing CT, but the level of evidence is still low 13.

Studies included in the previously mentioned review, evaluated a sole nutrition intervention of ONS enriched in omega-3 fatty acids (ONS-ω3) vs. placebo, an isocaloric diet, or an isocaloric ONS: they found significantly reduced weight loss and loss of fat free mass, and significantly increased skeletal muscle mass and lean body mass, QoL, and treatment tolerance in the groups receiving ONS-ω3.

In a recent pragmatic randomized controlled-trial conducted in 159 H&N cancer patients undergoing RT and CT + RT and receiving nutritional counseling, the use systematic use of ONS-ω3 resulted in better weight maintenance, increased protein-calorie intake, improved QoL and was associated with better anti-cancer treatment tolerance 45, with no additional costs for the healthcare system 46.

One limitation of most of the available studies on nutritional counseling and oral supplementation is their reliance on small sample size, different outcome measures, variable nutrition interventions, different timings and follow-up.

Of note, however, is a recent well-conducted multicenter study on 506 hospitalized cancer patients at nutritional risk, which showed that individualized nutritional support reduced the risk of mortality and improved functional and quality of life outcomes 47.

The optimal duration and timing of nutrition interventions should also be explored further, but the general impression that the early approach may be the most effective is gaining support 48.

## Nutritional Support in the Different Phases of the Disease

### Active Systemic Treatment

The often underdiagnosed unintentional weight loss and progressive skeletal muscle depletion are frequently present and worsen during CT and targeted therapies 22,23,49.

Regardless of cancer type, frailty level or age, patients may be similarly affected by malnutrition, 50-56, that is often linked with a range of different side effects, either due to the disease itself or the antineoplastic treatment, including oral mucositis, dysgeusia or smell alterations, fatigue, dyspepsia, nausea and vomiting. These events, by compromising adherence to therapies, may negatively affect outcome and limit the access to novel therapies.

Overall, the management of weight-losing cancer patients during active oncologic treatment relies on nutritional counseling, non-pharmacological (ONS and physical activity) interventions and pharmacological support, including artificial nutrition (AN) 10, 13-15, 23,57,58.

A proactive assessment of nutritional status is essential for selecting the most cost-effective intervention to apply: for example, oral nutritional support might reduce weight loss and hospitalization, and dronabinol may help to counteract nausea and vomiting or increase food intake. Ongoing randomized trials will make clear if treatment-naïve cancer patients at risk for malnutrition might also profit from an early, short-term, supplemental parenteral nutrition 59. Whether starving cancer cells may help increasing the activity of chemotherapeutic agents is currently unclear and prospective, well designed, randomized trials on caloric restriction or caloric restriction mimetics are missing 60. When tested under strict protocols, the preliminary clinical results are promising 61. However, due to the lack of solid clinical evidence, fasting and fasting-mimicking diets during active treatment are still not recommended, even in cancer types associated with a lower risk of malnutrition (e.g. breast and prostate cancer).

Novel preclinical data have suggested that a relationship between cell metabolism and susceptibility to immunotherapy may exist and uncontrolled studies have reported a correlation between BMI and efficacy of immunotherapeutic agents in several cancers 62-64.

Nevertheless, since conflicting data regarding any benefit from immunomodulators according to baseline BMI have been reported in different cancer types, this issue remains unresolved and the study of a more comprehensive or dynamic nutritional pattern has been suggested 65,66.

A secondary analysis of the data from the Swiss prospective, randomized-controlled, multicenter trial EFFORT compared the outcomes of a protocol-guided individualized nutritional support regime (intervention group) to standard hospital food (control group) in 506 patients with a main admission diagnosis of cancer and characterized by a broad spectrum of cancer sites, treatment types and disease severities. Individualized nutritional support reduced the risk of mortality and improved functional and QoL outcomes in cancer patients with increased nutritional risk, further supporting the inclusion of nutritional care in cancer management guidelines (47).

The efficacy of an early nutritional intervention was also highlighted in the study of Lu et al. (67), in which patients with metastatic esophagogastric cancer, who received an early interdisciplinary supportive care, provided by a team of gastrointestinal oncologists, nurse specialists, dietitians, and psychologists, integrated into standard oncologic care, was associated with an improved overall survival, compared with patients in the standard oncologic care-alone group (14.8 vs 11.9 months).

Overall, even if the above mentioned and other issues require further research-based evidence, we can affirm with confidence that prompt and strictly monitored nutritional support during oncologic active treatment is essential, in order to improve clinical outcomes and provide patients with the most innovative and effective therapeutic options.

Although activating a formal nutritional team in every hospital setting may be difficult, many successful examples may contribute in shaping a shared, cooperative awareness between patient communities and healthcare professionals 68.

The adoption of validated care pathways can ensure compliance with updated scientific recommendations, guarantee access to treatments backed by clinical evidence, contain clinical risks, rationalize expenditures, and increase equity.

### Surgical Setting

Nutritional care is a keystone for clinical outcomes in oncologic surgery.

For now, early oral feeding should always be the preferred nutrition mode after oncologic surgery, and this includes gastro-intestinal procedures 69.

The surgical operation causes a trauma that produces systemic inflammation, stress response and metabolic negative effects 70. The outcome of oncological surgery is not only related to the technical effectiveness of the surgical procedure itself, but also to the perioperative management. Lack of nutritional pre-operative screening and unnecessary post-operative fasting, raise the risk of underfeeding and surgical complications, especially after major surgery 71.

The perioperative management of surgical patients has been studied in depth in recent years and all these measures have been included in the Enhanced Recovery After Surgery (ERAS) programs 72: a scheduled perioperative pathway to minimize the surgical stress and improve the postoperative functional restoration 73. The ERAS protocol can be applied in many surgical fields such as colorectal surgery 74, esophageal surgery 75, pancreatic surgery 76, gynecological surgery 77 and many others 72.

Every patient undergoing major surgery should follow a personalized perioperative ERAS program, comprising scheduled steps, including nutritional care. Most of them are common to many different surgical specialties and procedures, such as:

- Preoperative nutritional screening with validated tools should be always performed before oncologic surgery, because severe under-nutrition has long been known to be detrimental to surgical outcome 78-80.

- Nutritional support should be started if the patient is malnourished or at nutritional risk before surgery. An appropriate nutritional support package should be applied during the hospital stay and following discharge 81. Nutritional interventions in the perioperative period includes counseling, ONS, EN and parenteral nutrition (PN) for all patients malnourished or at risk of malnutrition 69.

- In patients undergoing gastrointestinal surgery, the use of oral/enteral immunonutrition should be encouraged for a reduction in post-operative infectious complications 82.

- Preoperative fasting from midnight should be always avoided. Patients with no specific risk of aspiration can drink clear fluids until 2 hours prior to surgery and eat solid food until 6 hours before surgery 83.

- Oral feeding should be started as soon as possible after surgery, adapting oral intake according to type of surgery and individual tolerance. Special caution should be dedicated to elderly patients 84.

- Risk of postoperative ileus should be minimized and an opioid-sparing pathway should be applied, especially the use short-acting anesthetics during surgery and applying multimodal analgesia in combination with epidural analgesia post-operatively 85.

### Palliative Care

The role of nutritional support for oncology patients in palliative care is still a controversial issue.

However, in advanced cancer patients, preserving nutritional status may be an important goal also during the palliative care phase, because even when the disease can no longer be cured, patients may survive for several months or years. In this context, malnutrition may jeopardize performance status, QoL, tolerance to palliative treatments and survival.

AN can be integrated within a palliative care program when the risk of dying from malnutrition is greater than due to cancer progression 10,13. ESPEN guidelines suggest that EN should be considered first when the gastrointestinal tract is functional but oral food intake remains inadequate despite nutritional counseling and ONS, whereas, if EN is not sufficient or possible, PN is recommended 13.

Nevertheless, there are many factors that may adversely affect the provision of EN in these patients, e.g. high output ileostomy or intestinal fistulas, large bowel resections, and the presence of nutrition impact symptoms (abdominal pain, nausea, vomiting, diarrhea, or constipation due to peritoneal carcinomatosis). Also, ESPEN guidelines recommend home artificial nutrition (HAN), either EN or PN, in eligible cancer patients with persistent insufficient oral food intake or malabsorption 13.

However, in incurable patients nutritional support should be proposed considering the expected benefit on QoL and the potential benefit on survival 13. According to the principles of bioethics and ESPEN guidelines 13, the prescription of HAN should be discussed with the patient respecting his/her autonomy and, as also required by law, his/her choice or advance directive to refuse it. Concerning clinical appropriateness, HAN is not recommended in patients with short estimated life expectancy, severe organ dysfunction or uncontrolled symptoms, Karnofsky performance status <50 or Eastern Cooperative Oncology Group (ECOG) score ≥3, and in the presence of patient refusal 86.

Finally, an important question is whether there is evidence of a potential survival benefit due to home parenteral nutrition (HPN) in palliative cancer patients. Recently, a prospective study compared the survival of malnourished cancer patients in palliative care, eligible for HPN according to ESPEN recommendations, who received HPN with a homogenous group of patients, equally eligible for HPN, who did not receive HPN but artificial hydration for logistic reasons or because of patient refusal 87. Survival of the two groups showed a statistically significant difference in favor of the HPN group, which had a median overall survival three times higher than that receiving artificial hydration (4.3 versus 1.5 months, respectively).

## Critical Issues and Perspectives

Malnutrition in oncology is still under-diagnosed and untreated in an unacceptable proportion of patients 2-4 despite the clinical 45, 88,89 and economic 5,7 benefits resulting from early and appropriate nutritional support. The quality of nutritional cancer care in Italy, and to our knowledge throughout Europe 3, remains poor and far from the standards indicated by the international 13-15 and national 12 guidelines, and the Cancer Patients' Bill of Right for Prompt and Appropriate Nutritional Support 90.

One major issue is that the number of clinical nutrition units/services is inadequate and most oncology units are even devoid of dedicated dieticians. In Italy, according to the available data, there are around 50 clinical nutrition structured hospital units around the country, which is an insufficient number when compared to the number of oncology units (~330) 91 and these are unevenly distributed among the Italian regions.

In addition, clinical nutrition is an overlooked topic at every university teaching level, so that even the basic knowledge is lacking in young physicians and health care professionals.

Nowadays, the general education and training offered in clinical nutrition, which should be tailored to satisfy specific professional requirements, is qualitatively and quantitatively inappropriate in the majority of Western countries 92. Teaching of clinical nutrition is generally insufficient even within medical education, which is paradoxical, considering the relevance of nutritional issues in relation to both prevention and disease treatment 93,94.

The timing of nutritional intervention is fundamental in order to improve the therapeutic chances, with the “early approach” being the most effective 95,48. However, it is reasonable to think that not enough attention is still paid to this issue, as the most recent guidelines continue to be focused mainly on cachexia and advanced cancer patients 14,15.

Similarly, nutritional parameters are still not systematically evaluated and considered as potential confounders in outcome assessment and study design in clinical oncology trials.

Last but not least, disinformation remains a critical issue with regards to nutrition for cancer patients. Despite the lack of evidence-based data, hundreds of books and web sites still promote anti-cancer diets, fasting, fasting-mimicking diets and nutritional supplements, which are widely used without medical supervision 96. The autonomous use of dietary supplements should be discouraged, as they could interfere with oncologic treatments 97. They should only be prescribed by clinical nutrition specialists according to documented deficiencies and clinical conditions, and their efficacy and patients' compliance should be regularly monitored and reassessed.

Inadequate nutritional management for cancer patients should be considered ethically unacceptable and the lack of standard “high level” evidence cannot be a justification for overlooking nutritional care 96. However, providing additional evidence of its benefits from properly designed and sized clinical trials should be a key clinical objective in overcoming the barriers hampering the provision of proper nutritional support to cancer patients 2,10,98. Additional efforts for improving awareness on nutrition in cancer and counter the world of fake news is a priority and will require time and considerable effort, particularly at the institutional level, and for healthcare personnel. Cancer patient associations can play a fundamental role in sensitizing public opinion and Institutions in this regard. In order to avoid an excessive additional workload for oncologists and cancer care managers, it is reasonable to argue that the institution of multi-disciplinary teams or the inclusion of a clinical nutrition specialist in existing local tumor boards (at least for the cancer types associated with the highest nutritional risk [head&neck, gastrointestinal, lung]) is strongly recommended, as it can be beneficial to comprehensive patient management, by encouraging early referral and continuous nutritional support implementation and monitoring.

Another critical issue is the quality and the clinical relevance of the available nutritional studies in the oncologic field. Unfortunately, many trials report only nutritional outcomes rather than clinical outcomes (i.e. treatment response, toxicity, survival, QoL, etc.). Consequently, the effectiveness of nutritional support may result of limited interest for oncologists and the other health care professionals. Hence, the implementation of properly designed nutritional trials focusing on primary relevant clinical endpoints is key for the future development of clinical nutrition in oncology.

Among the topics of future studies, calorie and protein needs assessment for cancer patients should be of particular interest in light of the potential parallelism with the intensive care setting debate 99.

Preliminary experience adopting indirect calorimetry in the oncologic setting yield promising results 100 and further studies are ongoing. Waiting for the results, calorie and protein goals of patients with cancer should be still set according to the ESPEN guidelines 13, i.e. 25-30 kcal/kg and 1.3-1.5 g/kg, respectively.

From an economic point of view, quality improvement programs (QIP) involving nutrition support, such as the free provision of ONS at home, showed the potential for saving substantial resources 5,7,101. QIP in the nutritional care of cancer patients, should be developed and promoted, and reimbursement policies should be reassessed or modulated, accordingly.

The combination of nutritional support with personalized physical activity programs represents another promising and relevant issue, which deserves further exploration and implementation, in order to preserve muscle mass more effectively 102,103.

Finally, in the era of new technologies, telemedicine implementation may be extremely helpful for patient monitoring and support, leaving them safely at home, but its real applicability still requires proper assessment in the oncologic setting.

## Conclusions

Compared to the 2016 document, the updated Italian Intersociety WG for Nutritional Support in Cancer Patients makes additional practical recommendations, summarized in **Table 2**, and has identified some new areas/issues for possible improvement and implementation.

In order to implement these recommendations across the whole country, the WG will continue its work by establishing stable collaborations with national and international scientific societies/organizations, health authorities and academic institutions.

## Figures and Tables

**Table 1:** Nutritional counseling process in cancer patients

Nutrition Assessment and Reassessment:	• body weight assessment / changes / body composition; • biochemical data, medical tests and procedures; • energy, macro and micronutrient requirements; • actual food consumption (preferences and habits), and food and nutrition-related history; • estimated nutritional requirements; • cancer treatment side effects; • preferences, ethnicity, culture.
**Nutrition Diagnosis:**	• problems, difficulties and symptoms related to treatments that limit the consumption or absorption of nutrients; • obstacles to change (inconvenience, social problems, food preferences, lack of knowledge or time, costs).
**Nutrition Intervention:**	• definition of objectives; • meal set-up plan that emphasizes increasing meal frequency by distribution of foods to several small meals; • enriching dishes with energy- and protein-dense ingredients oral nutritional supplements; • food preparation and/or modifying of texture or nutrient content; • specific indication for mucositis and other symptoms, digestion (e.g. pancreatic enzymes) or absorption (e.g. slowing of rapid gastrointestinal transit), antiemetic, and other relevant conditions; • alliances with caregivers.
**Nutrition Monitoring/Evaluation:**	• monitoring and re-evaluation to determine if the patients has achieved, or is making progress toward, the planned goals.

**Table 2:** Summary of the Intersociety Italian Working Group for Nutritional Support in Cancer Patients updated recommendations

- Nutritional screening should be performed, preferably by nurses, using validated tools (NRS 2002, MUST, MST, MNA, PG-SGA) upon diagnosis, systematically repeated at each outpatient visit and within 48 hours since hospital admission.
- Patients at nutritional risk should be referred promptly for comprehensive nutritional assessment, possibly including the evaluation of body composition, and support to clinical nutrition services or medical personnel with documented skills in clinical nutrition.
- Patients with cancer types expected to affect nutritional status (head & neck, gastrointestinal, lung), advanced stage or treatment (high-dose chemotherapy, radical radiotherapy, major abdominal surgery or multimodal [either combined or sequential]) should be referred directly to clinical nutrition specialists for early comprehensive nutritional assessment, counseling/support and a strict monitoring program.
- Nutritional support should be initiated swiftly and targeted for each patient according to nutritional and clinical conditions, planned treatment and expected outcome. It should comprise nutritional counseling with the possible use of oral nutritional supplements and/or artificial nutrition (enteral nutrition, total or supplemental parenteral nutrition) according to the assessment and ensure the strict monitoring of spontaneous food intake, tolerance and effectiveness.
- Nutritional support and dietary modifications should aim to assist the maintenance or recovery of nutritional status by increasing or preserving protein and calorie intake. “Alternative hypocaloric anti-cancer diets” (e.g. macrobiotic or vegan diets), fasting and fasting-mimicking diets are not recommended.
- The autonomous use of dietary supplements should be discouraged. They should be prescribed by clinical nutrition specialists according to documented deficiencies and clinical conditions. Their efficacy and patients' compliance should be regularly monitored and reassessed.
- Every cancer patient undergoing major surgery should follow a personalized perioperative “Enhanced Recovery After Surgery” program that should comprise scheduled steps, including nutritional assessment and support.
- Nutritional support should be integrated into palliative care programs when the risk of dying from malnutrition is greater than from cancer progression, according to individual-based evaluations, quality of life implications, life expectancy and patients' will. Nutritional counseling aimed at alleviating nutrition-related symptoms should be provided to cancer patients receiving palliative care.
- Home artificial nutrition should be prescribed - even in the early phase if needed - and regularly monitored using defined protocols shared by all the healthcare professionals involved in patient care at institutional or, ideally regional/national level.
- Nutritional parameters should be always evaluated and considered as potential confounders in outcome assessment and study design in clinical oncology research.
- Adequately sized and designed clinical and cost-effectiveness trials, possibly involving cancer patient associations' representatives in the design process, are still needed in order to improve the evidence in favour of nutritional support in different care settings. The lack of standard “high level” evidence should not be a justification for overlooking nutritional care.
- The introduction of multi-disciplinary nutritional teams or the inclusion of clinical nutrition specialists in the existing local tumor boards (at least for the cancer types associated with the highest nutritional risk [head & neck, gastrointestinal, lung]) is strongly recommended.
